# Formative Study of Mobile Phone Use for Family Planning Among Young People in Sierra Leone: Global Systematic Survey

**DOI:** 10.2196/23874

**Published:** 2021-11-12

**Authors:** Emeka Chukwu, Sonia Gilroy, Kojo Addaquay, Nki Nafisa Jones, Victor Gbadia Karimu, Lalit Garg, Kim Eva Dickson

**Affiliations:** 1 Department of Computer Information System Faculty of Information and Communications Technology (ICT) Msida Malta; 2 United Nations Population Fund Country Office Freetown Sierra Leone; 3 National Secretariat for the Reduction of Teenage Pregnancy New England Freetown Sierra Leone

**Keywords:** young people, short message service, SMS, chatbot, text message, interactive voice response, IVR, WhatsApp, Facebook, family planning, contraceptives, Sierra Leone

## Abstract

**Background:**

Teenage pregnancy remains high with low contraceptive prevalence among adolescents (aged 15-19 years) in Sierra Leone. Stakeholders leverage multiple strategies to address the challenge. Mobile technology is pervasive and presents an opportunity to reach young people with critical sexual reproductive health and family planning messages.

**Objective:**

The objectives of this research study are to understand how mobile health (mHealth) is used for family planning, understand phone use habits among young people in Sierra Leone, and recommend strategies for mobile-enabled dissemination of family planning information at scale.

**Methods:**

This formative research study was conducted using a systematic literature review and focus group discussions (FGDs). The literature survey assessed similar but existing interventions through a systematic search of 6 scholarly databases. Cross-sections of young people of both sexes and their support groups were engaged in 9 FGDs in an urban and a rural district in Sierra Leone. The FGD data were qualitatively analyzed using MAXQDA software (VERBI Software GmbH) to determine appropriate technology channels, content, and format for different user segments.

**Results:**

Our systematic search results were categorized using Grading of Recommended Assessment and Evaluation (GRADE) into communication channels, audiovisual messaging format, purpose of the intervention, and message direction. The majority of reviewed articles report on SMS-based interventions. At the same time, most intervention purposes are for awareness and as helpful resources. Our survey did not find documented use of custom mHealth apps for family planning information dissemination. From the FGDs, more young people in Sierra Leone own basic mobile phones than those that have feature capablilities or are smartphone. Young people with smartphones use them mostly for WhatsApp and Facebook. Young people widely subscribe to the social media–only internet bundle, with the cost ranging from 1000 leones (US $0.11) to 1500 leones (US $0.16) daily. Pupils in both districts top-up their voice call and SMS credit every day between 1000 leones (US $0.11) and 5000 leones (US $0.52).

**Conclusions:**

mHealth has facilitated family planning information dissemination for demand creation around the world. Despite the widespread use of social and new media, SMS is the scalable channel to reach literate and semiliterate young people. We have cataloged mHealth for contraceptive research to show SMS followed by call center as widely used channels. Jingles are popular for audiovisual message formats, mostly delivered as either push or pull only message directions (not both). Interactive voice response and automated calls are best suited to reach nonliterate young people at scale.

## Introduction

### Sierra Leonean Context

Sierra Leone has a young population with an estimated 39.4% aged 15 to 35 years [1]. Pregnancy rates are high among adolescents. According to the 2013 Sierra Leone Demographic and Health Survey, overall 28% of adolescents aged 15 to 19 years have begun childbearing [2]. Among adolescents aged 15 to 19 years with no education, 46% have already started childbearing, which is more than double compared with 22% of those with secondary and higher education [2]. In Sierra Leone, cultural norms, low literacy, and limited access to information make young people prone to misinformation and inadequate knowledge of their sexual reproductive health and rights. Adolescents and young people hold many common myths and misconceptions about sexual reproductive health and family planning. A total of 36% of adolescents in the lowest wealth quintile have started childbearing compared to 14% in the highest wealth quintile [2].

Sierra Leone has low contraceptive use. Among the currently married, contraceptive use in young women aged 15 to 19 years is 8% [3]. Overall, urban dwellers have a high contraceptive prevalence of 26.6% compared with rural dwellers, with a prevalence rate of 13%. Myths, misinformation, and long-held traditional beliefs about contraceptives and long-term side effects on health, including infertility, are pervasive. The government is using different approaches to address these barriers, including awareness creation. Traditional contraceptive information dissemination methods use multichannel and engagement schemes as detailed in the national strategy to reduce adolescent pregnancy [4].

Funding from the United Nations Population Fund with the United Kingdom Department for International Development supports Sierra Leone’s Government in developing and deploying a mobile-enabled family planning information awareness program. Researchers are increasingly using formative research to understand the best entry strategy, aid planning, and design interventions. Similarly, qualitative research is accepted in health programs and research [5] and is mainly used in formative research. Digital health, “the systematic application of information and communications technologies, computer science, and data to support informed decision-making by individuals, the health workforce, and health systems to strengthen resilience to disease and improve health and wellness” [6], often benefits from formative research–based design.

### Mobile Technology

Mobile technology (mHealth) can democratize access to critical sexual reproductive health and family planning information by reducing barriers such as stigma and fear often experienced by young people. Digital-enabled awareness can help eliminate traditional economic, geographic, and literacy constraints. Evidence shows that mobile technology, when appropriately applied, can help improve knowledge and awareness of end users [7] and caregivers [8]. Mobile phone ownership is emerging as a measure of socioeconomic status for underserved regions of the world [9]. Evidence from Kenya, with similar socioeconomic demographics as Sierra Leone, shows that mobile phone ownership is directly proportional to educational status, wealth, and having fewer children [9]. In Sierra Leone, technology is playing a pervasive role in several sectors, including the health care sector. Sierra Leone has an estimated 79 connected mobile SIM per 100 population teledensity [10]. The global pandemic occasioned by COVID-19 has shown the impact of technology in general and mHealth, particularly for information and services [11]. Emerging technologies beyond mHealth are increasingly being adopted for health and social service delivery [11]. There is an increasing surge in mobile apps for service delivery, particularly maternal and child health [12]. Similarly, policy makers are prioritizing digital technologies for strategic health care improvements [13].

### Study Objective

This study is formative research to aid the design of a technologically appropriate and culturally relevant mHealth intervention in Sierra Leone. The specific objectives of the formative research were as follows:

- Understand the global trends on the mobile phone for family planning information dissemination
- Explore how young people in Sierra Leone use mobile phones
- Understand the needs and barriers to family planning information and services
- Gain information to support the design of a technology appropriate and culturally relevant mHealth intervention

The sections that follow detail the approach, main findings, and study recommendations.

## Methods

This formative study involved a systematic scholarly literature search and review and focus group discussions (FGD).

### Literature Review

We systematically searched 6 databases considered representative of both family planning (contraceptives) and mobile technology. We conducted a systematic search including relevant published papers from 2000 to 2019. We used the search keywords in Table 1 and their extensions.

**Table 1:** Search terms and returned results.

Search terms	Scopus	IEEE^a^	EBSCO	PubMed	SpringerLink	Web of Science	Total
SMS AND family planning	35	2	126	36	283	25	507
Chatbot AND family planning	2	0	1	0	6	0	9
Interactive voice response AND family planning	7	3	4	6	55	5	80
mHealth AND family planning	66	3	179	144	141	60	593
WhatsApp AND family planning	1	1	1	2	44	1	50
Facebook AND family planning	24	7	25	14	371	32	473
Twitter AND family planning	10	4	20	9	200	8	251
Hotline AND family planning	31	1	28	59	257	12	388
Call center AND family planning	124	15	9	114	106	1	369
YouTube AND family planning	7	1	1	6	172	0	187
SMS AND contraceptive	23	1	113	30	386	15	568
Chatbot AND contraceptive	1	0	1	0	3	0	5
Interactive voice response AND contraceptive	7	0	6	7	63	5	88
mHealth AND contraceptive	44	2	127	74	104	34	385
WhatsApp AND contraceptive	1	0	2	0	36	1	40
Facebook AND contraceptive	19	0	60	24	345	13	461
Twitter AND contraceptive	3	0	10	11	195	2	221
Hotline AND contraceptive	19	0	31	71	211	5	337
Call center AND contraceptive	45	0	11	94	78	2	230
YouTube AND contraceptive	2	0	10	5	161	5	183
Total	471	40	703	706	3217	226	5363

^a^IEEE: Institute of Electrical and Electronics Engineers.

**Figure 1:**
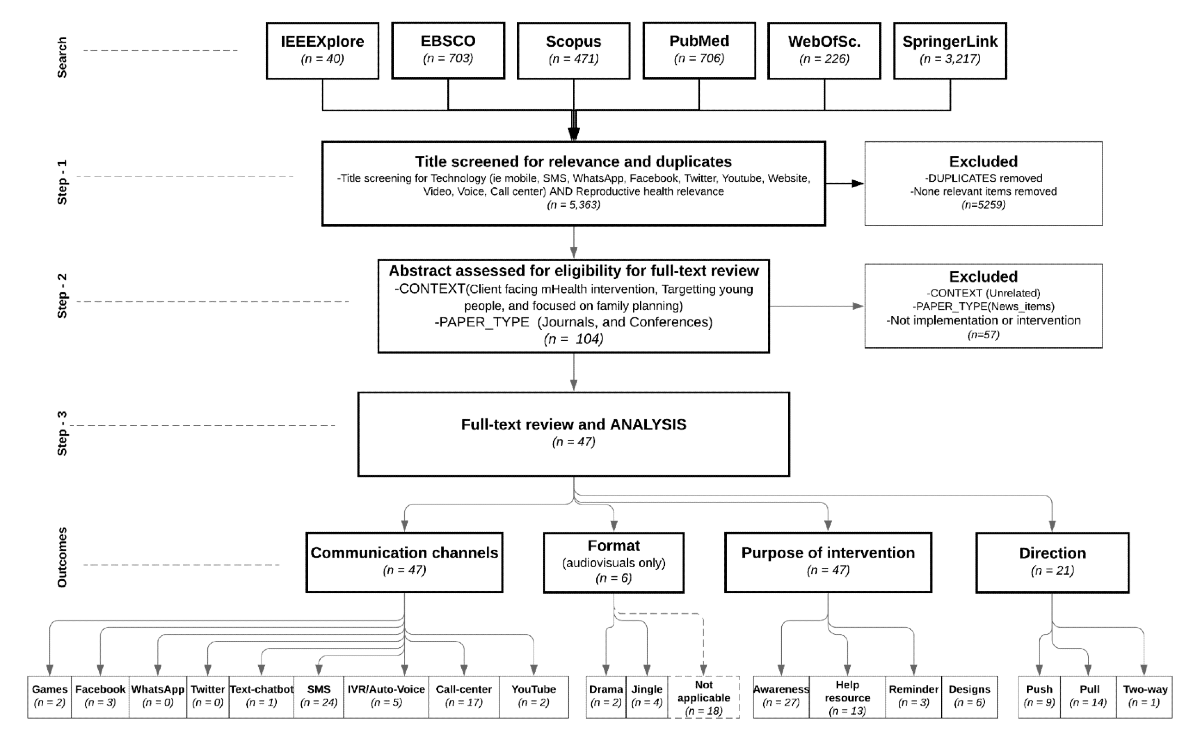
PRISMA flowchart on our systematic review of global mobile health research focused on family planning.

The search identified 5363 papers. After deduplication and removal of nonrelevant items through title screening, 104 papers remained. Titles were screened for inclusion if they reflected a combination of both technology and reproductive health. For technology, the keywords of interest were mobile, SMS, WhatsApp, Facebook, Twitter, YouTube, website, video, voice, call, call center, or their app synonyms. All returned results were included in an Excel (Microsoft Corp) research information template through this process. The 104 article abstracts were then reviewed for context and paper type. Our context of interest was citizen-facing mHealth interventions for family planning. Paper types included were conference publications and journal articles. Only 47 papers were included after screening the 104 abstracts and excluding 57 publications for no implementation or not being related to our context. The number of items deemed eligible for full-text review came to 47 papers. See Figure 1 for our PRISMA (Preferred Reporting Items for Systematic Reviews and Meta-Analysis) process to review the 47 papers.

We used the Grading of Recommended Assessment and Evaluation (GRADE) scheme to categorize the outcome of our full-text review and analysis into communication channels, audiovisual content format, intervention purpose, and message flow direction [14].

### Focus Group Discussions

To better understand and engage stakeholders and end users (young people) who may be affected or impacted or who may influence mHealth intervention outcomes, we conducted a series of FGDs. The interactions helped gather feedback on how young people use mobile phones and their internet use habits, phone ownership, and expense patterns.

#### Participant Recruitment

We conducted the FGDs in 2 districts, one predominantly urban (ie, Freetown–Western Area Urban) and the other mostly rural (Moyamba). Moyamba district also has high early marriage with a median age of marriage being 17.5 years, below the national average, 18.2 years, and below the age of consent [15]. Also, 36.8% of girls in Moyamba have begun childbearing by age 18 years [16]. This is high compared to overall, with 28% of adolescents aged 15 to 19 years having begun childbearing. Target participants in both districts were purposely selected for each group targeting 5 to 10 participants in each group. Within each district, 4 groups were targeted as follows:

- Junior and senior secondary school students (male and female) to target end users with basic literacy, aged 14 to 18 years
- Youth corps members (male and female) to target end users with postsecondary education, aged 19 to 24 years
- Community learning center (CLC) participants to target females aged 14 to 24 years for nonliterate end users
- Village welfare committees, consisting of community members with authoritative knowledge of young people in the community

**Figure 2:**
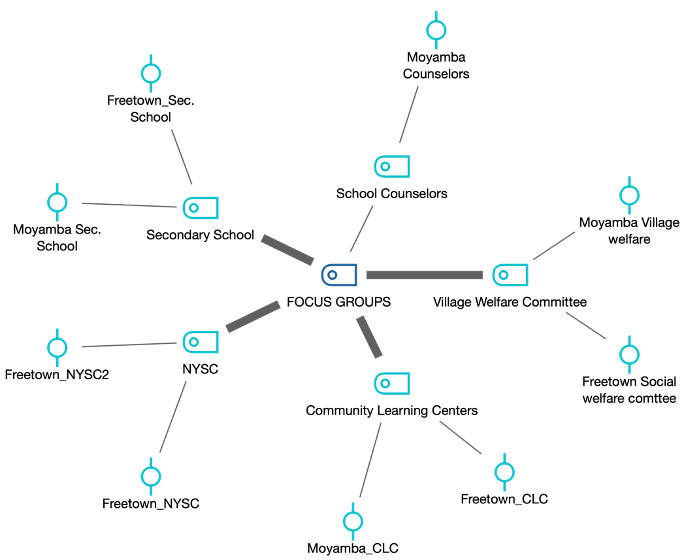
Focus group segments.

CLCs are centers where out-of-school girls learn and participate in vocational activities. Conversely, the village welfare committee is a variable member committee that oversees the community members’ welfare. Their work spans health, education, and environmental welfare issues, and they generally represent the parents and gatekeepers for young people in their communities. The National Youth Service Corps (NYSC) group are graduates undergoing a 1-year national service. The group of school counselors provides counseling services to secondary schools. In all, we conducted 9 FGDs with target participants from NYSC, CLC, secondary school, village welfare, and school counselors, as shown in Figure 2. Table 2 further illustrates the number, distribution, and characteristics of participants in the 9 FGDs in Freetown and Moyamba districts. The semistructured discussion guide was pretested with 5 participants: a nurse, 2 secondary school pupils, and 2 college students prior to the FDGs.

#### FGD Approach

Discussions were conducted in the Krio language for all 9 groups. Each session started with an introduction to the purpose of the visit and discussion using the research information template as in the guide introduction. Informed consent was sought from participants, each having a copy of the consent form and the facilitator reading the text in the Krio language. Each consenting participant was required to sign a consent form without providing personal information. The transcripts were transcribed following key themes in the semistructured questionnaire immediately after each group session. The only quantitative data are the aggregate number of participants and their aggregate demographics.

**Table 2:** Characteristics of focus group discussion participants.

Days 2019	Discussion sites	Participants	Gender	Age (years)	District
Aug 19	Pretesting FGD^a^ tool	5	Mixed	23-25	Western Urban
Aug 21	Secondary school (junior and senior)	7	Mixed	18-24	Western Urban
Aug 22	National Youth Service Corps	4	All female	>18	Western Urban
Aug 21	Community learning center	10	All female	14-19	Western Urban
Aug 22	Village welfare committee	7	Mixed	>23	Western Urban
Aug 29	Secondary school (junior and senior)	10	Mixed	14-19	Moyamba
Aug 28	Community learning center	8	All female	14-23	Moyamba
Aug 29	Village welfare committee and nurses	10	Mixed	>24	Moyamba
Aug 28	School counselors	11	Mixed	>24	Moyamba
Sep 4	National Youth Service Corps	10	Mixed	>18	Western Urban

^a^FGD: focus group discussion.

### Data Coding and Analysis

We identified and organized the FGD transcripts into key themes and coded using MAXQDA Analytics Pro (version 20.2, VERBI Software GmbH) [17]. The transcripts were coded by FGD, demography, phone ownership, phone type, phone use, phone top-up, phone expense, internet subscription, internet use, sexual and reproductive health (SRH) information access, language preference, current SRH medium, preferred SRH medium, comfort discussing SRH, and preferred SRH content. We then performed grouping using constant comparative analysis of these themes adapted from grounded theory proposed by Chun Tie et al [18].

### Ethics Approval

Approval was sought and obtained to conduct the study from Sierra Leone’s Ministry of Health and Sanitation’s Ethics and Scientific Review Committee on August 13, 2019. We did not collect individually identifiable information during the FGDs.

## Results

This section reveals the study findings and builds on existing global research frameworks and insights from Gonsalves et al [19] and McCarthy et al [20]. Findings from our systematic literature search are presented first followed by results from the FGDs.

### Literature Review Findings: The Global State of Mobile for Family Planning

Based on our GRADE evaluation, reviewed papers’ intervention channels include games, Facebook, WhatsApp, chatbots, SMS, interactive voice response (IVR), automated voice messages, call centers, and YouTube, as illustrated in Table 3. Call center and SMS are by far the most used channels from our findings. No study intervention used custom app, WhatsApp, or Twitter as an intervention channel. Similarly, the purpose of the majority of these interventions in Table 3 is for contraceptive awareness, followed by those providing resources for contraceptive information and service. A few were for reminders, design, or research purposes.

**Table 3:** Article distribution showing channels and purpose of intervention.

Channel type	Games	Facebook	WhatsApp	Twitter	Chatbots	SMS	IVR^a^/autovoice	Call center	YouTube	Video
Purpose of intervention	—^b^	—	—	—	—	—	—	—	—	—
Awareness	[21,22]	[23,24]	—	—	—	[20,23,25-39]	[37,40-42]	[35,36,43-45]	[32,46]	[32]
Reminders	—	—	—	—	—	[47,48]	[40]	—	—	—
Help resource	[22]	—	—	—	[49]	[30,49,50]	[42]	[42,51-58]	—	—
Feasibility and design study	—	[59]	—	—	—	[60-62]	[58]	[63]	—	—

^a^IVR: interactive voice response.

^b^Not applicable.

IVR mobile content delivery channel has been used for maternal health [64], postabortion care [40], and family planning [58]. The earliest was conducted in Cambodia in 2013 [40]. Although most interventions did not indicate the language, those that did were IVR-delivered contents, and the language of content delivery was the local language (not English). SMS interventions for family planning improved consumer knowledge by 14% (from 9.9% to 18.2%) compared to a control group with limited knowledge [27]. No SMS intervention for family planning studies discussed the language of delivery. Social media has also been used in other regions for family planning–based demand generation like the peer-led safe sex Facebook group in China [59]. Other demand generation interventions include serious games to enhance sexuality education for young adolescents in Hong Kong [21]. Serious games use virtual reality–enabled games with engaging family planning information. A video-based mobile technology intervention has equally shown promise among adolescents in the United States [32]. In Kenya, the Shujaaz multimedia platform used various channels ranging from comic radio programs, a Facebook campaign, and SMS [23]. Most of the papers reviewed did not provide details on the content structure.

Given the complexity in designing audiovisual interventions, we further analyzed and categorized the audiovisual intervention papers into message format and message direction. The SMS intervention similarly has a 160-character limit with directions push, pull, or 2-way interactive. Audiovisual messages were delivered in drama or jingle style as shown in Table 4. Call centers were mostly configured so that people call in. Some interventions used multiple channels [32,35-37], while some were designed for more than one purpose. The intervention by Smith et al [40], for instance, is for awareness and reminders, as shown in Table 3.

**Table 4:** Article distribution showing audiovisual formats and message direction.

	Message format			Message direction		
	Drama style	Jingle style	Pushed to recipient		Pulled by recipient	Two-way
IVR^a^/autovoice	[58]	[37,40-42]	[37,40-42]		—^b^	[58]
Call center	—	—	[35,42,48,51,53]		[36,40-45,52,54-57,63]	—
YouTube and videos	[32]	—	—		[32]	—

^a^IVR: interactive voice response.

^b^Not applicable.

### FGD Findings

#### Mobile Phone Ownership, Subscription, and Use Patterns

**Figure 3:**
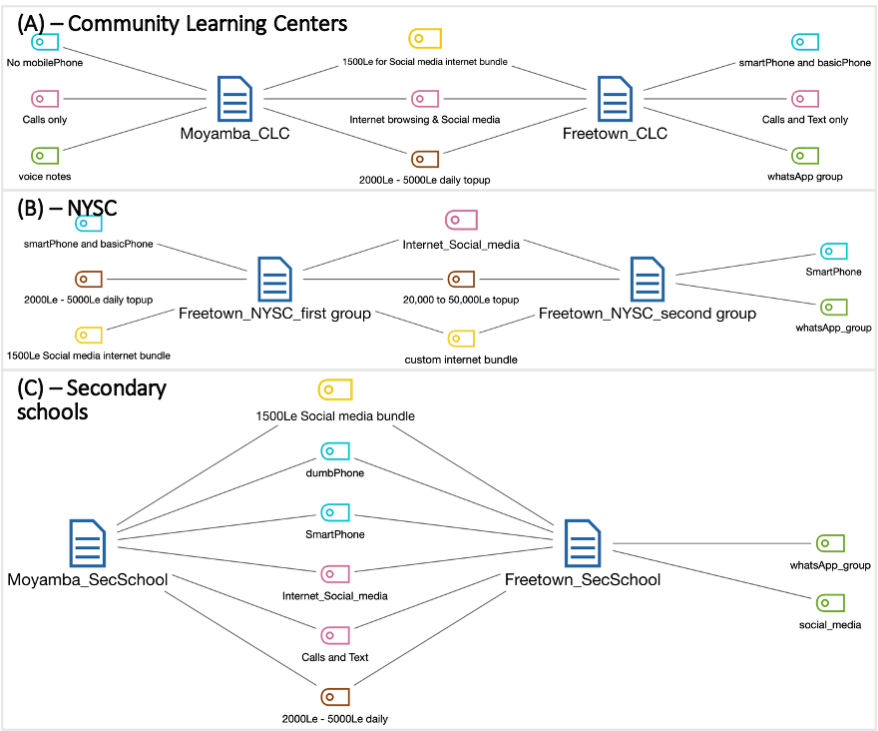
Phone ownership, use, top-up, internet subscription, and internet use by focus group.

No participants in the CLC group in the Moyamba rural district had a mobile phone, although they indicated sharing with their family members (see blue in Figure 3A). The CLC group in the Freetown urban district comprises participants who own smartphones and basic mobile phones (blue). Those who have used smartphones indicated subscribing exclusively to the social media bundle (yellow) among the CLC groups. Figure 3A shows these; both CLC groups credit or top-up their phones with 5000 leones (US $0.50) or less to make calls (brown). The voice call credit (top-up) subscriptions were intermittent and far between for the CLC participants, especially those with basic phones.

I sell top-up, and young people mostly buy 2000 leones.... Out of every 10 purchases, 8 of them will be 2000 leones.

Village welfare committee respondent

Conversely, as shown in Figure 3B, all participants in the NYSC group had phones. In the second NYSC group, all participants had smartphones, most participants subscribed to a higher internet plan, and only one participant subscribed to the social media bundle. The majority of young people received their phones as gifts from parents, relatives, or friends.

Some girls can get their phone from boyfriend.

Moyamba district respondent

I bought mine from a shop to pay back in installments.

Moyamba district respondent

I can’t use social media bundle because one can’t do anything with it except Facebook and WhatsApp.

NYSC respondent

You cannot even watch YouTube videos with the social media bundle.

NYSC respondent

The NYSC participants’ daily call credit use was less consistent but much higher than most other groups.

I will credit an average of 20,000 leones [US $2.10] to 50,000 leones [US $5.24] every now and then, and refill when exhausted.

NYSC respondent

[I] refill with 4000 to 5000 leones [US $0.42 to $0.52] daily.

NYSC respondent

I use unlimited internet plan that cost 500,000 leones [US $55.56] because of my business. That is what works for me because other internet plans exhaust before the end of the month.

NYSC respondent

Figure 3C illustrates the case for secondary school pupils.

#### Mobile Phone Use and Language

Participants in all groups were unanimous that WhatsApp group discussion and texts happen mostly in English, while video and audio messages or discussions are conducted mainly in Krio.

I only call with my phone and we speak Mende or Krio language...

Moyamba CLC respondent

Calls are almost exclusively in Krio or local dialect. When asked if she sends SMS with her phone, she responded, “No.”

Until you understand and write English, you cannot understand and write Krio.

School counselor respondent in the village welfare committee

**Figure 4:**
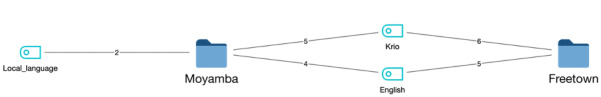
Language preference distribution.

The qualitative analysis of responses from both districts, as shown in Figure 4, indicates that young people in Freetown preferred either English or Krio language, while those in Moyamba sometimes prefer any of their local dialects (Mende or Temne) in addition to English and Krio. The FGDs revealed that the NYSC group uses WhatsApp the most, followed by Facebook and then banking or mobile money apps.

#### Young People’s Information Habits

**Figure 5:**
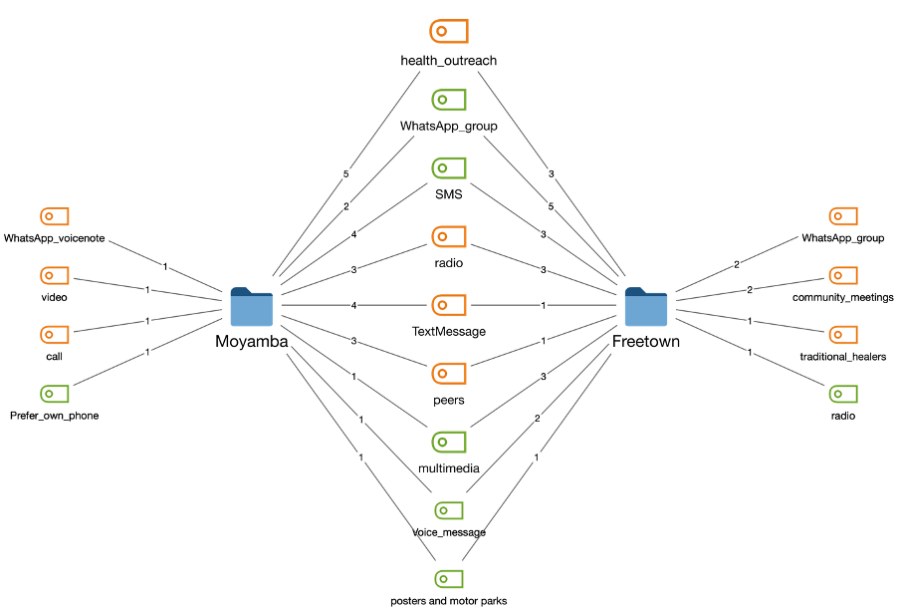
Distribution of current and preferred sexual and reproductive health information medium by district.

In Figure 5, the color amber is for the current information medium, and the color green is for the preferred information medium based on focus group response analysis. The weighting on the lines indicates how many groups indicated each option from the transcript. WhatsApp group, SMS, voice message, multimedia message, and posters at motor parks featured prominently.

People from Marie stopes always bring their buses with fitted speakers to parks and market places around to promote family planning.

Moyamba CLC respondent

I subscribed for daily health information for 2500 leones per week by dialing *931# on the Orange network. When there is no credit balance, the SMS messages are delayed and get delivered as soon as I top-up.

School counselor cohort respondent

Some of the health topics discussed include rape, early pregnancy, and early marriage. Peers are the medium predominantly used for information clarification by secondary school pupils.

Awuko news sends regular health information from the Ministry of Health by SMS.

Moyamba CLC respondent

A WhatsApp group is considered an effective avenue for message dissemination by this group. Secondary school pupils discuss their sexual health with their parents. Participants who own smartphones consistently showed a preference for WhatsApp as a message receipt channel compared to call or SMS channels.

I think SMS may be better for disseminating information compared to calls because you can refer to it again after reading the first time, and you can show others unlike calls.

NYSC respondent

The NYSC group indicated they have more trust in messages received via SMS, although they could not explain why they think so.

#### SRH Information, Myths, Misconceptions, and Parents

**Figure 6:**
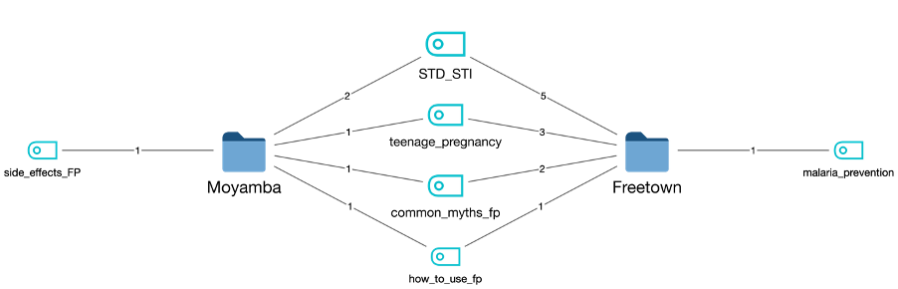
Distribution of preferred sexual and reproductive health information medium by district.

Young people identified information on sexually transmitted diseases and sexually transmitted infections as what they want to hear about the most. This is followed by a preference for information on how to prevent teenage pregnancy (see Figure 6). One issue identified was that parents and young people are worried about the long-term effect of contraceptives. A Moyamba CLC respondent told a story she heard from a friend about “a girl whose hand was cut off because the implant caused cancer.” When asked if she knew the girl in question, her response was “no.” Another issue that a nurse in the community welfare group tried to solve is one where “the parents force young girls to take out already inserted implants.”

The nurse explained that this is rampant and that health workers discourage this practice by increasing the cost of removal to 40,000 leones (US $4.44). As a result of these reasons, some participants believe that some mothers fabricate myths to stop their girls from taking these contraceptives for fear of promiscuity.

## Discussion

Our literature review, coupled with the FGDs, provided global and local insight and context to help us decide on an appropriate strategy for an mHealth for family planning intervention.

### Understanding Global Trends in Mobile for Family Planning Information Dissemination

Our systematic review revealed health information had been delivered through mobile for maternal health [64], postabortion care [40], and family planning [27]. An SMS campaign in Kenya, for example, helped improve recipients’ knowledge by 14 percentage points [27]. The 1-way pull or push message directions were predominant. Bidirectional intervention content was featured only once in the literature. Communication channels were Facebook, YouTube, SMS, voice calls, and call center. Most research study interventions were on SMS and call center, and none on WhatsApp or Twitter. We found from aggregated studies that the educational background, language, and phone ownership pattern directly correlate with the choice of a delivery channel. Only literate users, for instance, can access text-based content. Low-literate young people will be better reached through voice-based delivery like IVR and person-manned call centers. Low-literate users may not be reachable with multimedia like videos due to their often limited access to smart devices. Evidence of implementation or impact of mobile demand generation initiatives for family planning is still lacking in Sierra Leone.

Related to our systematic survey, a study surveyed the 2 popular app stores for Android and iOS and found 5276 and 877 custom apps, respectively, designed for maternal and child health [12]. These apps did not appear in our systematic literature search of scholarly databases. There are 2 main reasons why this may be so: (1) they may not have met the study inclusion criteria or (2) they may not have had scholarly contributions at the time of survey and writing.

### Exploring How Young People in Sierra Leone Use Mobile Phones

Mobile phone ownership appeared to increase with educational attainment, as a pattern seems to have emerged when we compared out-of-school young people at CLC or secondary school pupils or NYSC members. Ownership of a mobile phone is now considered an item of immense importance, and those who do not have one indicated sharing with their family and friends. To buttress this point, a participant told the following story.

My friends will always make fun of me for not having a phone. When I was opportune to volunteer for an NGO and was paid a stipend, I did not save any of the money. I went straight to the shop where I bought the android phone...to avoid the shame.

Freetown CLC respondent

Despite this high interest, many young people still do not own their mobile phones, possibly due to funding constraints. Sharing mobile phones will often reduce the privacy of the content on that mobile. Participants who use or own smartphones prefer WhatsApp and Facebook as the primary communication channels.

Conversely, basic mobile phone owners are constrained to performing essential phone functions: voice call and text messages. The majority of smartphone users outside the NYSC cohorts used the social media bundle, allowing Facebook and WhatsApp access only. This may limit how much rich multimedia content can be delivered to these young people. Participants with a smartphone use a social media bundle subscribed to daily. The expense pattern described indicates that even participants who subscribe to a social media bundle can miss out on any multimedia rich intervention even if they may be interested as a result of funding constraints. Options available will be to provide data to them for this or deliver content that is not dependent on internet data but available through the telecommunication network infrastructure.

### Understanding the Need and Barriers to Family Planning Information and Services

The users at the bottom of the phone ownership pyramid understand Krio and their other local languages. These users do not generally write for communication. This means that those who cannot read in English cannot read in Krio, which corroborates the earlier finding. In deciding an intervention strategy, the user’s phone type is only one of many factors to consider. There are other trade-offs to the final decision, including available resources, intended time to market, and technical implications. When users are asked without regard to their affordability and current spending habits, massive multimedia channels such as videos, social media, Mobi-sites, and mobile apps then become viable channels. Among the low-literate young people, there is a large proportion who still do not have a mobile, and they can be reached via radio and television.

### Gaining Insights to Support the Design of Technologically Appropriate and Culturally Relevant mHealth Interventions

From the literature review and FGDs, it was determined that to reach young people who have basic phones and are less literate, an audio-based system that would need to be delivered to a basic phone was necessary. The 3 options were either a call center, prerecorded automated calls, or an IVR. On the other hand, literate users with basic phones can be reached with text messages in the form of SMS. However, this would only reach a limited group, leaving out the most vulnerable. This has been proven to be effective in other countries [27]. Moreover, it is relatively less expensive and less technically involved once the message content has been developed.

### Conclusion

The high incidence of teenage pregnancy and low contraceptive prevalence rate among young people necessitated the formative research. The study would help inform the design of an mHealth intervention as one component of broader efforts to delay sexual intercourse among young people and increase demand for family planning among those who are sexually active. The formative research helped show that globally, SMS followed by call center are the 2 widely used messaging channels. Audiovisual message formats were either drama or jingles delivered as push or pull only message directions (not both). For interventions that indicated message direction, only 1 of 21 implemented 2-way messaging; others were either push or pull 1-way messaging.

Solutions like automated SMS, call centers, IVR, chatbots, Facebook, and YouTube have been used worldwide. For scalability, the priority channels are SMS, call center, or IVR because they can reach users with basic mobile phones who make up most phone users among young people [10]. However, the volume of content delivered per time is limited for the same cost compared to other media-rich channels like videos, animations, and audio. Although not widely researched, multimedia channels like WhatsApp, Facebook, and video-based mobile apps are an emerging area appropriate and engaging for literate and affluent users. Young people’s education level and socioeconomic disposition affect their mobile phone ownership, phone use patterns, and access to health information. These characteristics are relevant for designing appropriate mHealth interventions in Sierra Leone and elsewhere.

## Abbreviations

CLC: community learning center
FGD: focus group discussion
GRADE: Grading of Recommended Assessment and Evaluation
IVR: interactive voice response
mHealth: mobile health
NYSC: National Youth Service Corps
PRISMA: Preferred Reporting Items for Systematic Reviews and Meta-analysis
SRH: sexual and reproductive health
